# Large-Scale Distribution of the European Seahorses (*Hippocampus* Rafinesque, 1810): A Systematic Review

**DOI:** 10.3390/biology11020325

**Published:** 2022-02-18

**Authors:** Cataldo Pierri, Tamara Lazic, Michele Gristina, Giuseppe Corriero, Mauro Sinopoli

**Affiliations:** 1 Department of Biology, University of Bari Aldo Moro, 70125 Bari, Italy; cataldo.pierri@uniba.it (C.P.); tamara.lazic@uniba.it (T.L.); giuseppe.corriero@uniba.it (G.C.); 2 National Research Council of Italy IAS—Institute of Anthropic Impacts and Sustainability in Marine Environment, 90149 Palermo, Italy; 3 Stazione Zoologica Anton Dohrn, Department of Integrative Marine Ecology, 90149 Palermo, Italy; mauro.sinopoli@szn.it

**Keywords:** syngnathids, PRISMA, long-snouted seahorse, short-snouted seahorse, *Hippocampus* distribution, ecological assessment

## Abstract

**Simple Summary:**

Populations of many marine species are threatened by a number of interacting factors, including anthropogenic activities, climate change, and biodiversity loss. The assessment of the conservation status of such populations relies heavily on several types of data, such as large-scale geographical and ecological distribution. Seahorses are charismatic fish sensitive to environmental pressures, and according to the IUCN directive 95/2020, they should be considered a model for environmental quality assessment. As in many other areas, the data on seahorse ecological distribution in Europe are scattered, patchy, and mainly focused on small-scale studies. Therefore, we undertook a systematic review using the PRISMA protocol to identify the current knowledge status, detect gaps, and propose future research priorities. We analyzed 32 years of published studies and described the distribution of *Hippocampus guttulatus* and *H. hippocampus* across 176 sites in the Atlantic Ocean, Mediterranean Sea, and Black Sea as a function of habitat, depth, and degree of confinement. The applied method evidenced the overall lack of a detailed habitat description in published studies. Seahorse conservation would benefit from an analytical description of habitats, such as data on the depth, nature of the substrate, and associated biological communities, as well as the use of a standardized habitat classification system, such as formally recognized EUNIS habitat codes.

**Abstract:**

Human pressures on marine ecosystems have caused extensive degradation of marine habitats and several local extinctions. Overexploitation and destructive fishing practices are responsible for biodiversity loss in many coastal ecosystems. The definition of conservation programs in marine fish requires comprehensive knowledge on large-scale geographical distribution, while considering distribution/abundance patterns in relation to key environmental variables. Due to their life-cycle traits, the two European seahorses (*Hippocampus guttulatus* and *H. hippocampus*), as with other congeneric species, are particularly sensitive to the effects of anthropogenic activities and habitat changes. However, information on the ecological distribution of these two species is scattered, patchy, and mainly focused on small-scale studies. In this paper, we followed an international standard protocol for systematic reviews (the PRISMA protocol) to provide a detailed assessment of the two species’ geographical distribution in relation to the environmental characteristics. According to the 134 analyzed studies, *Hippocampus guttulatus* is more common in confined areas, while *H. hippocampus* is found in marine shelf waters. With several interspecific differences, seagrasses were the most used holdfasts of both species. The EUNIS codes (European nature information system) referring to a specific and unique habitat were discussed as a potential tool for defining the ecological distribution of the two species. The obtained results and their future implementation could help plan conservation actions.

## 1. Introduction

Worldwide, human pressures on marine ecosystems have caused extensive degradation of marine habitats, and by impacting many communities, have also caused several local extinctions [1,2]. Indeed, overexploitation of fish and other seafood resources, coupled with destructive fishing practices, pollution, introductions of alien species, and climate change, are mainly responsible for biodiversity loss in coastal ecosystems [3]. In this scenario, species with unique life-cycle traits (such as low swimming capacities, mate fidelity, lengthy parental care, and high site fidelity) and close trophic relationships with local communities seem much more sensitive to environmental changes, and this could especially refer to seahorses [4,5,6,7,8].

The success of specific conservation actions will rely heavily on the quantity and quality of data available on the large-scale geographical and ecological distribution, as well as on the environmental drivers that underlie the ecological dynamics of communities [9,10]. The relationship between the distribution/abundance patterns and features of habitats is well known, and features such as depth, bottom type, and physical characteristics [11] are correlated to the spatial distribution of many species [12,13]. Unfortunately, comprehensive knowledge of these data in marine fish are available in only a limited number of cases, such as commercially important species, whereas for most other species, even if of conservation interest, there is very often an information gap.

Seahorses are charismatic fish considered flagship species of the conservation efforts [14,15], the populations of which tend to be patchily distributed and occur at low densities worldwide [4]. These fish are characterized by sedentary behaviour, low swimming capacities, and small home ranges [4,8,16,17,18]. Furthermore, seahorses live in vulnerable coastal habitats that, together with their unusual life cycle traits, make them sensitive to the effects of anthropogenic activities and habitat change [4,6,7]. 

The two seahorse species *Hippocampus guttulatus* and *H. hippocampus* have a wide geographic range extending across most of Europe and North Africa, including the Atlantic Ocean, Mediterranean Sea, and Black Sea [19]. Like other congeneric, both species are listed on Appendix II CITES (Appendix II) and the IUCN Red List where they are classified as “Data Deficient” at a global level [14,15]. Declines in several populations across Europe have been recently reported [5,17,20,21,22]. Although the exact causes remain unknown, there are some indications of a decrease in suitable habitats [20] or even illegal trafficking [20,21]. Such a status for the two species, indeed, indicates the urgent need for specific conservation actions to preserve populations. However, due to their cryptic nature and sedentary behavior, seahorses are difficult to survey, and this poses serious challenges to their conservation [23]. 

Estimating the conservation status of populations could benefit from knowledge about their distribution [24], and could help identify species hotspots [25]. A systematic review of the available literature could be useful to achieve this goal, as systematic reviews synthesize evidence, identify gaps in the literature, and can suggest future lines of research [26]. In recent years, several reviews on seahorses [27,28] and a specific one on two European species [23] have been published. Indeed, a literature search may help gain a more complete picture of the species distribution, demographics, and thus conservation status [29]. In the present paper, we used the methodology of systematic reviews, well established in ecology and conservation [30,31], to summarize 32 years of published studies on *H. guttulatus* and *H. hippocampus* so as to provide an increasingly fine and detailed assessment of the geographical and ecological distribution in relation to the environmental characteristics. By providing data on spatial distribution and its correlation with environmental features, the results of this study will help with better conservation of the two seahorse species. 

## 2. Materials and Methods

The systematic review was carried out according to the orientations of Preferred Reporting Items for Systematic Reviews and Meta-Analyses (PRISMA) [32], which is used as a guide for study selection, screening, and eligibility. Studies were identified using several search engines, including Elsevier’s Scopus (www.scopus.com (accessed on 1 November 2021)), Clarivate Web of Science (www.webofknowledge.com (accessed on 1 November 2021)), and Google Scholar (scholar.google.com (accessed on 1 November 2021)). The bibliographic search included peer-reviewed literature, theses, books, and other related scientific reports published between 1989 and 2021. Several combinations of keywords were used to identify relevant publications: “*Hippocampus guttulatus*”, *Hippocampus hippocampus*”, “*Hippocampus ramulosus*”, “seahorse”, “long-snouted”, and ”short-snouted”. Reference lists of publications were also used as bibliographic sources. Potentially relevant papers were read in full, and information and data that were relevant for this review were extracted. Studies considered duplicates and those that included animals raised in captivity were excluded from the analysis. In order to represent the comprehensive spatial distribution of seahorses, studies without clear toponymic references were used in the case of data-poor countries (e.g., the Maghreb and North/East Africa). Furthermore, regarding the same areas, additional searches were made to recover reliable sources (i.e., peer-reviewed publications, scientific reports, and congress communications) by using digital platforms not included in the initial PRISMA strategy. When available, information on study type (biodiversity/other), target species (*H. guttulatus* or *H. hippocampus*), year of record, study site, country, sea (Mediterranean Sea, Atlantic Ocean, or the Black Sea), coordinates, confinement (marine environment or lagoon), abundance, density, sex (abundance/density of males, females, and juveniles), habitat (e.g., *Posidonia oceanica* or sandy bottom), and depth were collected. Several publications did not report the entire set of required information (such as studies on commercial catches or checklists); in such a case, we used the available data, which were combined together and expressed as percentages. The only exceptions were EUNIS habitat codes [33]; if possible, they were obtained by comparing the described habitat characteristics with the analytical descriptions of the EUNIS codes at the highest possible level (level 4).

## 3. Results

**Figure 1:**
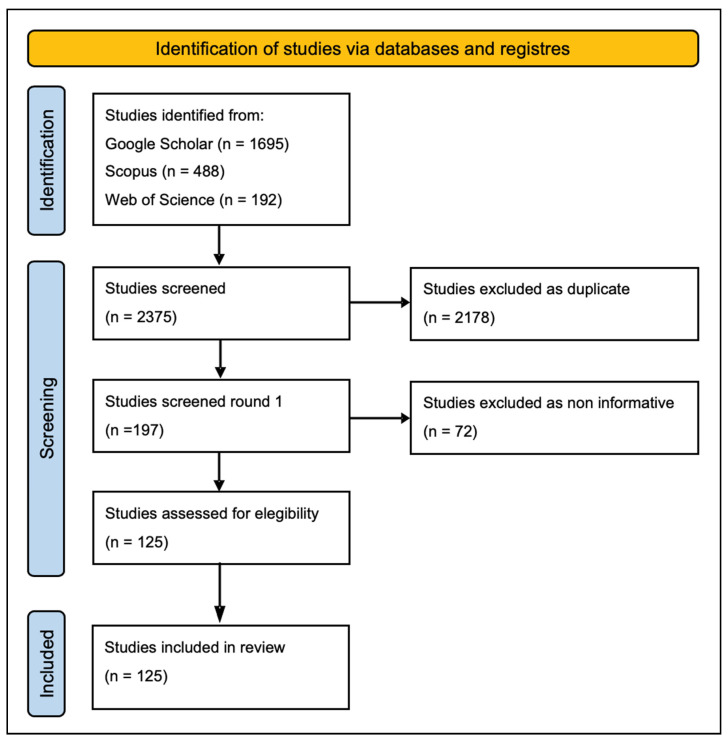
Flow chart detailing the process of identification, screening, and eligibility of references for the systematic review.

The PRISMA search strategy found 2375 preliminary studies. After validation procedures and the removal of duplicates and non-informative studies, 125 studies were chosen (Figure 1). An analysis of the bibliographic sources from alternative search engines (not included in the initial PRISMA strategy) and referred to the data-poor regions revealed six peer-reviewed publications, two technical reports, and one congress paper, accounting for 134 studies used for the distribution analysis.

### 3.1. Research Trends and Publication Metrics on Seahorses

**Figure 2:**
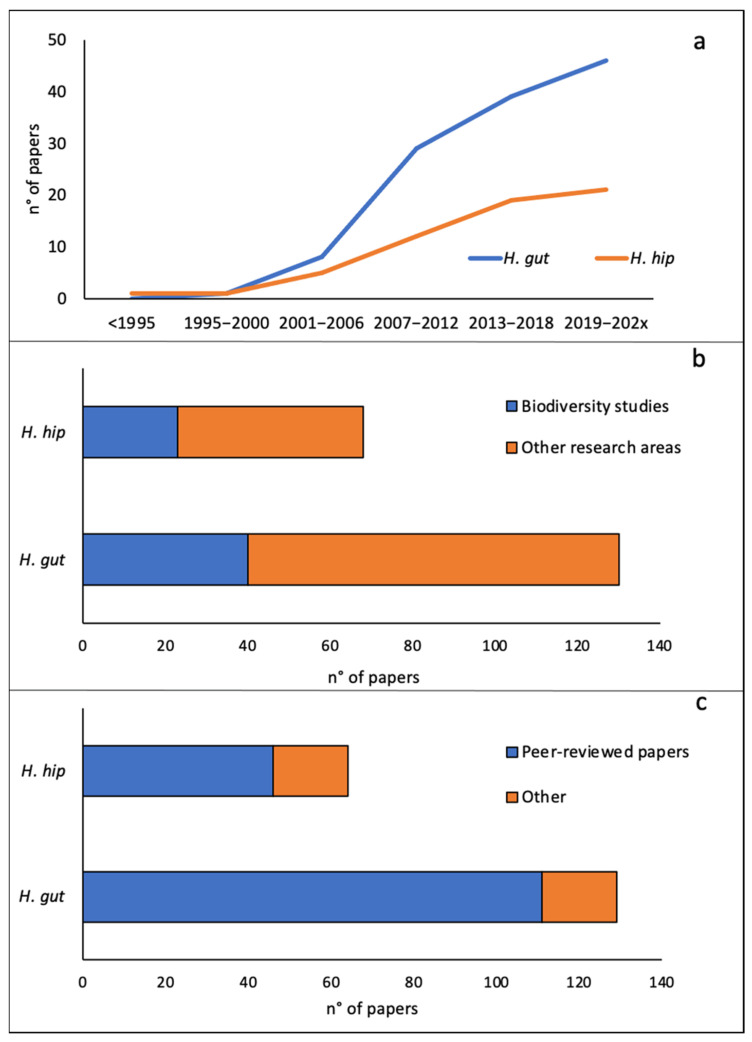
(**a**) The number of publications on *H. guttulatus* (in blue) and *H. hippocampus* (in orange) per year. (**b**) The number of publications on *H. guttulatus* and *H. hippocampus* per research topic. (**c**) The number of publications on *H. guttulatus* and *H. hippocampus* per publication type.

The first scientific publication on European seahorse species dates back to 1989, although the first observation dates as far back as 1948. The number of scientific publications increased only in the mid-2000s, and the number of published papers reached a maximum between 2011 and 2015 (Figure 2a). Most papers focused on *H. guttulatus* and referred to many scientific areas (Figure 2b). Both species were rarely reported in biodiversity studies (Figure 2b). Regarding the type of publications, peer-reviewed papers were the most abundant for both species (Figure 2c), while the other types (e.g., theses, books, and technical reports) accounted for 10% of publications. 

### 3.2. Geographical and Macroecological Distribution

**Figure 3:**
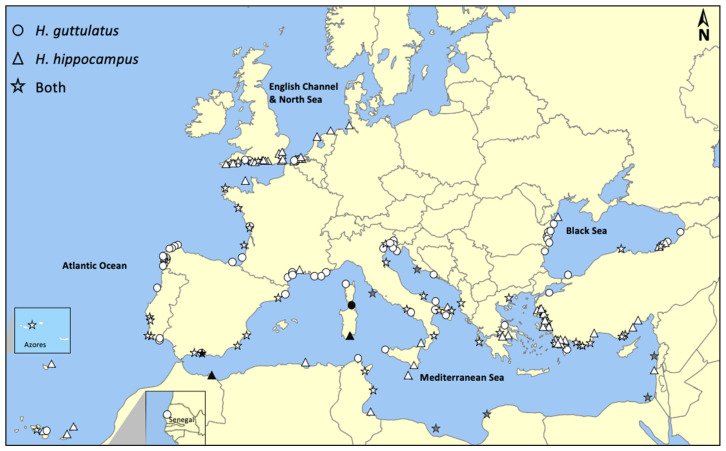
Distribution map of seahorse species. White symbols indicate sites found following the PRISMA strategy. Black symbols indicate sites found through alternative search engines. Gray symbols indicate the presence of seahorses without a clear indication on the exact position.

According to the PRISMA strategy, publications referred to 167 sites across the entire species distribution range, including the Northern Atlantic Ocean (namely the English Channel and the North Sea), the Atlantic Ocean, Mediterranean Sea, and Black Sea (Table 1 and Figure 3). Most of the recorded sites were in the Mediterranean Sea (*n* = 82), followed by the Northern Atlantic Ocean (*n* = 34), Atlantic Ocean (*n* = 33), and Black Sea (*n* = 18). Sites were located in 22 countries, and more than 65% were related to the following five countries: Turkey (*n* = 39), Spain (*n* = 24), the United Kingdom (*n* = 18), Italy (*n* = 16), and France (*n* = 13). An analysis of the alternative search engines revealed nine additional sites in the Mediterranean Sea, located in Italy (*n* = 3), Croatia (*n* = 1), Libya (*n* = 2), Morocco (*n* = 1), Egypt (*n* = 1), and Lebanon (*n* = 1). 

In the Mediterranean Sea, the two species frequently co-occurred at the same sites (approximately 40%). Seahorses in other areas were distributed independently with similar values (Table 1). In the Atlantic Ocean and the Black Sea, the two species co-occurred at a low number of sites (36% and 11%, respectively), with *H. guttulatus* being the most reported species. On the contrary, in the English Channel and the North Sea, *H. hippocampus* was more frequently observed, and the number of sites at which the species co-occurred was also low (18%). 

**Figure 4:**
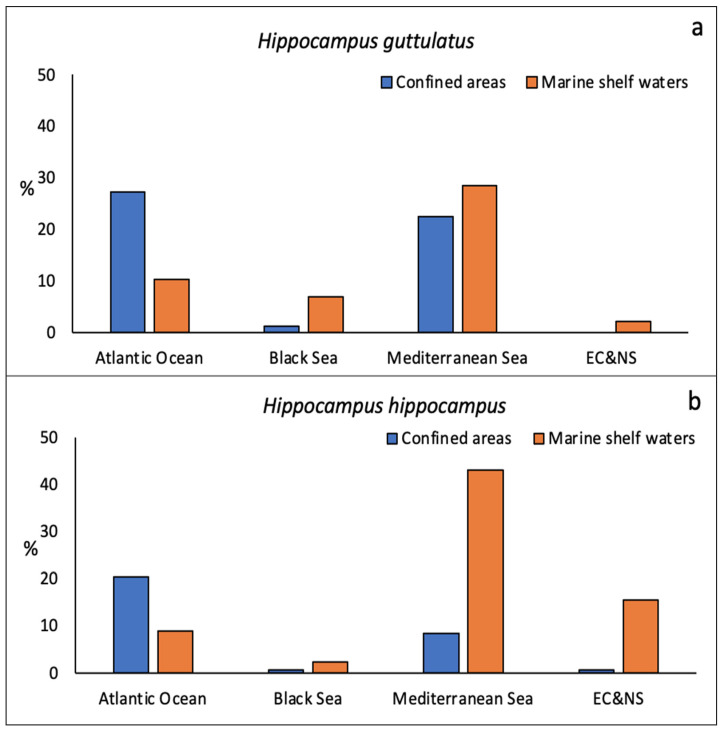
Percentage distribution of seahorses concerning geographical area and the degree of confinement for (**a**) *H. guttulatus* and (**b**) *H. hippocampus*. EC&NS = English Channel and the North Sea.

The two seahorse species had different confinement preferences among the different seas (Figure 4a,b). In the Atlantic Ocean, both species were mainly recorded in confined areas (lagoons, estuaries, or semi-enclosed bays). In other seas, on the contrary, both species were most frequently described in marine shelf areas. This trend was especially evident for *H. hippocampus* in the Mediterranean and Northern Atlantic Ocean (Figure 4b); in the Mediterranean, the distribution of *H. guttulatus* in confined and open sea areas was almost overlapping.

### 3.3. Ecological Distribution 

**Figure 5:**
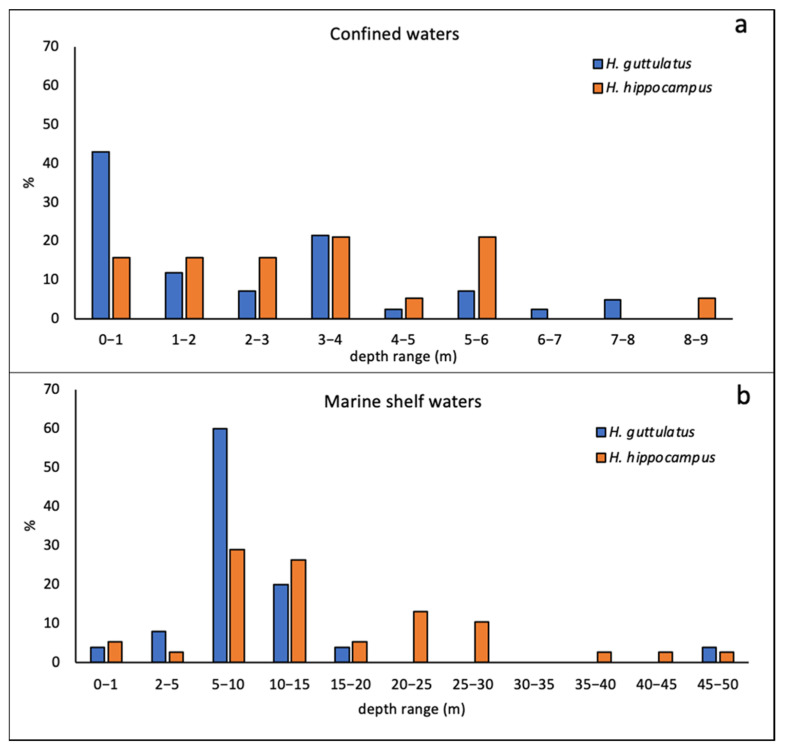
Percentage depth ranges (meter) of the two species in confined (**a**) and marine shelf (**b**) environments.

Focusing on the considered habitat parameters, a greater amount of information was present for substrate preferences (124 publications) rather than depth (111 publications). Preferred categories of water depth varied according to the species and confinement. In confined areas (Figure 5a), both species were mostly found from the surface up to six meters of depth, with weak differences between species. Indeed, although *H. guttulatus* was usually reported at depths from 0 to 1 m, *H. hippocampus* was more frequently described at depths ranging from 4 to 6 m. In marine shelf waters (Figure 5b), the peak of *H. guttulatus* reports was found at depths between 5 and 10 m, with a low number of records at depths greater than 20 m. *H. hippocampus*, however, was most described at depths ranging from 5 to 30 m, and the number of specimens observed at depths greater than 30 m was low.

**Figure 6:**
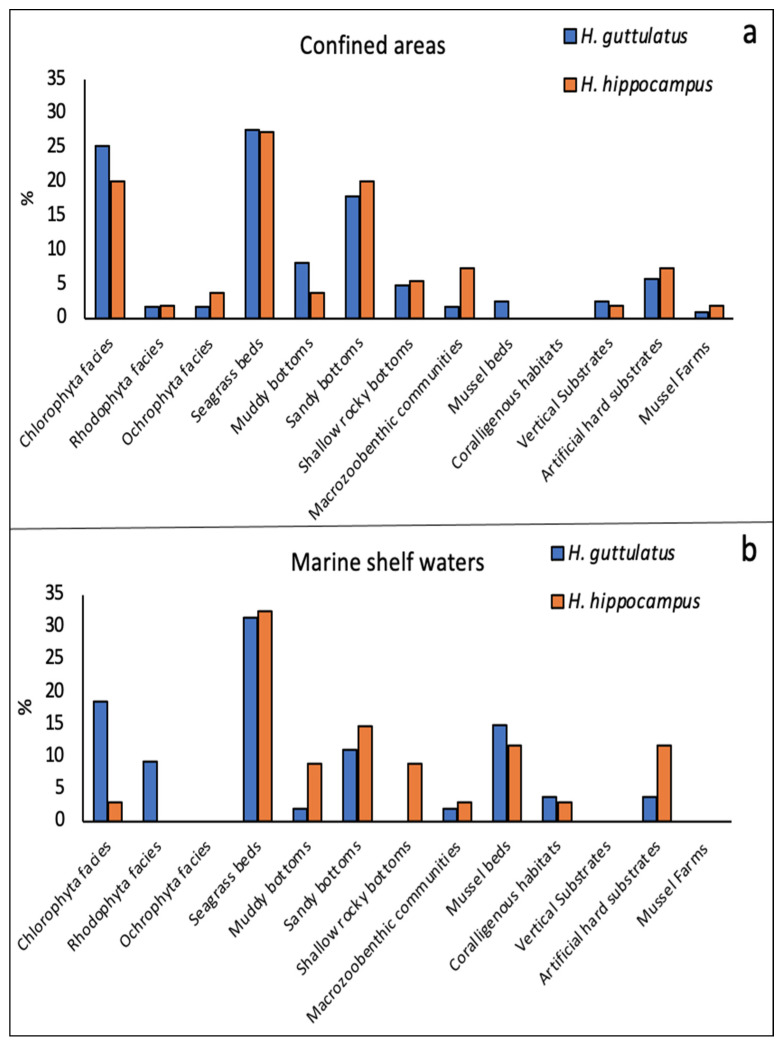
Percentage distribution of the two species among habitats in confined (**a**) and marine shelf environments (**b**).

Concerning habitats, seahorses were found in 24 different habitat types among all sites (Figure 6a,b). However, in confined environments, the two species were reported in more habitats (*n* = 21) than in the marine environment (*n* = 15). At both confined and marine sites, the two species were most frequently reported in seagrass beds. *Hippocampus guttulatus* also showed high preferences for Chlorophyta facies and sandy bottoms at both types of sites, and additionally for Rhodophyta facies and mussel beds in marine shelf areas (Figure 6b). *H. hippocampus*, on the contrary, was rarely reported in association with algae, but was more frequently found on phanerogams and other substrates, including shallow rocky and muddy bottoms, almost always sharing the same ecological distribution of *H. guttulatus*.

**Figure 7:**
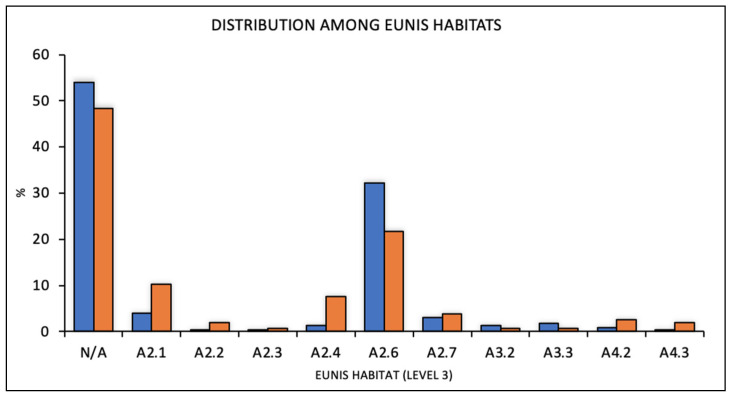
Distribution of the two species among the habitats classified according to the EUNIS (European Nature Information System) classification system.

Publications with data sufficient for the correct classification of habitats according to the EUNIS classification system (Figure 7) permitted the identification of three types of EUNIS level 2 habitats, including A2 (Littoral sediments), A3 (Infralittoral rock and other hard substrata), and A4 (Circalittoral rock other hard substrata), and ten types of EUNIS level 3 habitats (for the interpretation of these habitats, see EUNIS 2020 habitat classification). Following this classification, most of the publications reported seahorses in the habitat of Littoral sediments dominated by aquatic angiosperms (Level 3: A2.6; Level 2: Littoral sediments). With increasing depth and transition from infralittoral to circalittoral habitats, the number of seahorse reports decreased with a greater frequency for *H. hippocampus* at greater depths. 

## 4. Discussion

One of the fundamental challenges at the forefront of conservation biology is to understand the ecological and spatial distribution of sensitive species and their demographic dynamics. This challenge becomes greater when attempting to develop conservation strategies for data-poor species with patchy and scattered information. Seahorses are a paradigmatic case of data-poor marine species. In that sense, the systematic review could compensate for the lack of specific large-scale studies by synthesizing the available information.

Seahorses are considered flagship species in several fields of conservation biology [19] and have been recently claimed to have an important role as indicators of environmental quality [34]. The issues facing seahorses, including habitat degradation and loss, target fisheries, and by-catch, are indeed major concerns in marine conservation, and the fact that the global IUCN status of *H. guttulatus* and *H. hippocampus*, as for many other seahorse species, is Data Deficient [14,15] indicates the need for a specific implementation of current knowledge to improve their conservation status. The decline of many seahorse populations, coupled with their unique appearance and life history, has generated considerable interest among many scientists, with an increase in the number of publications in the last decades. However, despite the increasing number of studies and reported declines of up to 80% of the initial population abundances [22], both species are still poorly considered in national directives and laws regulating their collection (with some exceptions; in the UK, for instance, both species are protected by the Wild-Life and Countryside ACT and are among the UK Biodiversity Action Plan priority species) [14,15]. The necessity for the correct classification of the species status on regional and international levels has also been highlighted by the IUCN resolution 95 (WCC-2020-Res-095-EN the Conservation of seahorses, pipefishes and seadragons). Characterization studies containing both ecological and autecological information could be useful to achieve this scope. However, seahorses lack in-depth characterization at a global scale, as most ecological studies refer to small-scale assessments [7,20,27,35]. Using the methodology of systematic reviews, the present study brings together all available information present in the scientific literature, thus representing the most exhaustive and up-to-date assessment of the geographical and ecological distribution patterns of *H. guttulatus* and *H. hippocampus*. Moreover, the study proposes the use of EUNIS codes, the main comprehensive pan-European hierarchical habitat classification system, as an important tool for designing networks of protected areas, monitoring, and management planning. 

By reporting 32 years of records and 176 sites across the entire distributional range, this research contributed to strengthening the ecological and geographical assessment of European seahorses. *H. guttulatus* and *H. hippocampus* were recorded across the entire distributional area [14,15,19], and the number of occurrence localities of the two species reported here is greater than that of previously published datasets [4,19,23]. The data collected during this systematic review confirmed the trend of increased scientific attention and, although these charismatic fish have been studied for more than 30 years, the research intensification began only recently, when several European research groups simultaneously focused their attention on these animals. However, most studies analysed in our review were focused on reproductive biology or physiological traits, while a smaller number were related to autecology and population dynamics. The focal point on these biological aspects was also common among other seahorse species, as unique life cycle traits provide a significant opportunity to expand our understanding of reproductive ecology in animals in general [27].

The important role of confined environments, such as lagoons and estuaries, was particularly evident in the Atlantic, probably because they offer shelter from strong wave motions and winter storms [36]. Furthermore, the tendency to occupy shallow habitats could explain why many seahorses in the Atlantic Ocean were found in confined areas. In the Mediterranean Sea, these fish seemed more equally distributed between the two environments, although *H. guttulatus* showed slightly higher preferences for confined areas, while *H. hippocampus* was more frequently found at marine sites, probably because of the greater water depths, which seem preferred by the species [7]. Although in agreement with previous small-scale studies, it should be pointed out that such findings could be an artefact of site-specific population traits. Indeed, the results are highly dependent on the research activities in specific areas with locations where seahorses were already known to be present or abundant being more studied, and this could have limited our ability to detect ecological patterns. 

Regarding ecological distribution, *H. guttulatus* and *H. hippocampus* can be found in a variety of habitats with different degrees of complexity, but prey abundance seems an important factor in determining habitat selection [17,34]. However, according to the available information, the most reported seahorse habitat in both marine shelf and confined areas were marine phanerogams. Seagrasses are the preferred habitat of many temperate syngnathid species [4], probably because they maximize prey density and capture efficiency, and could help fish with low swimming capacities avoid predators [4]. In confined areas, however, seahorses are also reported on incoherent (sandy or muddy) bottoms and algal beds, supporting site-specific studies [7,20,23,37,38]. Some seahorses have been recorded grasping artificial structures, highlighting their important role in the population dynamics of seahorses [18,37]. It is known that artificial structures are a suitable habitat for seahorses, probably because they host rich and diversified fouling communities, which can contribute to the complexity of the system by providing additional microhabitats, food, and hiding places [7,35,39].

In the present research, we attempted to standardize habitats of occurrence by applying the European codification system used in many ecological studies. The EUNIS habitat classification is a comprehensive pan-European system used to facilitate and harmonize the description and collection of data using analytical criteria. There are two advantages of using this classification: first, its use of widely accepted habitat types recognized by the scientific community, and second, it is a reference point for the development of indicators and environmental reporting [40]. When considering the high variability of habitats over the entire seahorse distribution area, the use of standardized codes could be essential to map habitats of their occurrence, support conservation strategies, and environmental assessment. However, the performed systematic review revealed a substantial deficit in data availability, as only half of the selected literature contained an entire set of the required information on environmental features, which somewhat hampered our analysis. According to the available data, most seahorses, and especially *H. guttulatus*, have been recorded in the habitat of Littoral sediments dominated by angiosperms (EUNIS code A2.6), confirming the results of site-specific studies on arbitrarily defined habitats [7,17,23,28,34,35]. 

Following the results of the present systematic review and previous site-specific studies [14,15,19], both species have a wide and mainly overlapping geographic range extending across most of the Atlantic Ocean, Mediterranean Sea, and Black Sea. However, parts of the two species distribution areas appear poorly represented according to the peer-reviewed bibliographic sources, although numerous unpublished expert opinions and observations have testified the presence of seahorses. The presence of populations, their abundance, and other survey information, for instance, seem incomplete at the southern limit of the seahorses’ range, including broad areas along the African coast (e.g., Algeria, Egypt, Libya, and Morocco). In line with accepted principles of literature searches for systematic reviews, this approach uses specific bibliographic databases to identify adequate literature. However, the search engine can prevent finding all pertinent records, as literature is often found outside of the required bibliographic databases and might involve websites or online repositories that typically require specific data searching and browsing. Therefore, keyword-based research on specific databases may miss items potentially relevant to the research question. Furthermore, although journal articles are usually easily identifiable via database searches, other research items such as research reports and conference papers are often not [34]. Indeed, when the literature analysis was expanded in specific geographic areas, the search produced new records of seahorse occurrence, mainly referring to ichthyofauna checklists and unpublished sources (such as technical reports of scientific projects and congress abstracts). Assessing material published on the Internet represents a challenge, given the vast amount of information, lack of standard indexing, and controlled vocabulary, but it can make important contributions to a systematic review [41] as much research is unpublished or not disseminated through peer-reviewed, commercial media [42,43]. The results of the systematic review presented in this paper revealed some important findings that would not have been apparent without a search on these non-standard sources. Indeed, the use of additional research platforms provided a greater definition of seahorse occurrence data. However, this review revealed that many bibliographic sources did not report the information useful for georeferencing seahorses, such as toponyms or geographical coordinates of sites. Such constraints imposed a challenge to the comprehensive review of seahorse distribution. Although usually excluded as non-informative in the screening process, in the case of countries with no data, we decided to insert the generic report on the distribution map (Figure 3) in order to present the information as complete as possible.

The outcome of the systematic review will depend not only on the ability of search strategy to locate relevant data, but also on the quality and quantity of those sources and the information they contain. When dealing with systematic reviews, an aspect that should not be underestimated is the identification of the weight that should be given to each record in relation to the research effort, as collected information could seem unbalanced—abundant and detailed in the countries where the research teams are historically involved, while rare or scanty in other regions, possibly because of a lack of research interest or funding to study the species. The results of this review were probably influenced by the presence of research groups that actively studied the two species on a local scale. Furthermore, the lack of studies following the same populations over time, thus providing historical series of repeated observations, rendered any systematic representation of the information incomplete. This applies to a series of sites at which seahorses were claimed to be present, but there were no indications of their past or contemporary abundance nor eventual population fluctuations through time. In the case of several sites, such as Mar Piccolo di Taranto (Southern Italy) and Ria Formosa (Southern Portugal), there was repeated information confirming a constant seahorse presence, albeit with numerical fluctuations. According to some anecdotal evidence, several populations were abundant in the past, but have now completely disappeared, as is the case with Marsala lagoon (Sicily). This situation is probably shared by other populations, but the available literature does not contain sufficient information that would permit the assessment of abundance changes, thus rendering any restoration and conservation actions difficult.

The conservation of seahorses should be a priority for ecological, biological, and economic reasons, as well as for their intrinsic value [28]. Although there are a relatively high number of papers on the two European seahorses that have been published in recent years, some aspects of seahorse ecology and biology seem poorly represented. The assessment of the conservation status is influenced by data availability and the possibility of their effective use [44]. Indeed, according to the FAIR principles, information needs to be findable, accessible, interoperable, and reusable in order to be used as a basis for decision-making when converted into knowledge [45]. If conforming to recognized and standardized protocols, uniform characterization of the information would allow for the application of artificial intelligence tools for the search, interpretation, and cleaning of large amounts of data. This review highlights advances in our understanding of seahorse distribution, but perhaps more importantly, it highlights important gaps to be filled. Therefore, we suggest that future research should be oriented to locate and characterize hitherto unknown populations, especially in poorly studied areas, which would be valuable for understanding the distribution and ecology, and could help at preserving the two seahorse species.

## Figures and Tables

**Table 1:** Occurrence (and co-occurrence) sites of the two species in the Northern Atlantic Ocean (the English Channel and the North Sea), Mediterranean Sea, and the Black Sea according to PRISMA (*n* = 167) and alternative search engines (*n* = 9).

	*H. guttulatus*	*H. hippocampus*	Both Species
English Channel and North Sea	5	23	6
Atlantic Ocean	17	4	12
Mediterranean Sea	28	25	38
Black Sea	13	3	2

## Data Availability

In light of recent threats posed by illegal harvesting, the authors believe that they should not publish the exact list and related locations of sites of seahorse occurrence. However, the data will be made available to anyone with a scientific or political interest after a justified request to the corresponding author.
